# Contemporary evidence on colorectal liver metastases ablation: toward a paradigm shift in locoregional treatment

**DOI:** 10.1080/02656736.2021.1970245

**Published:** 2022

**Authors:** Yuan-Mao Lin, Reto Bale, Kristy K. Brock, Bruno C. Odisio

**Affiliations:** a Department of Interventional Radiology, The University of Texas MD Anderson Cancer Center, Houston, TX, USA;; b Interventional Oncology-Microinvasive Therapy (SIP), Department of Radiology, Medical University Innsbruck, Innsbruck, Austria;; c Department of Imaging Physics, The University of Texas MD Anderson Cancer Center, Houston, TX, USA

**Keywords:** Colorectal cancer, liver metastases, percutaneous ablation, liver resection

## Abstract

Image-guided percutaneous ablation techniques represent an attractive local therapy for the treatment of colorectal liver metastases (CLM) given its low risk of severe complications, which allows for early initiation of adjuvant therapies and spare functional liver parenchyma, allowing repeated treatments at the time of recurrence. However, ablation does not consistently achieve similar oncological outcomes to surgery, with the latter being currently considered the first-line local treatment modality in international guidelines. Recent application of computer-assisted ablation planning, guidance, and intra-procedural response assessment has improved percutaneous ablation outcomes. In addition, the evolving understanding of tumor molecular profiling has brought to light several biological factors associated with oncological outcomes following local therapies. The standardization of ablation procedures, the understanding of previously unknown biological factors affecting ablation outcomes, and the evidence by ongoing prospective clinical trials are poised to change the current perspective and indications on the use of ablation for CLM.

## Introduction

Despite notable advances in the clinical application and outcomes of liver ablation for the treatment of colorectal cancer liver metastasis (CLM), substantial gaps in knowledge still exist in respect of its head-to-head comparison to surgical resection. According to international guidelines [1], resection is currently considered the local therapy of choice for patients presenting with resectable CLM. The National Comprehensive Cancer Network guidelines have recommended ablation alone or ablation used in conjunction with resection as long as all visible diseases can be eradicated [2]. Such predilection is predicated by historical data showing that, when compared to ablation, resection is associated with improved overall survival (OS), disease-free survival, and reduced local recurrence rates [3–12]. Notwithstanding, recent understandings on patient selection, tumor biology, ablation technology, and image guidance, along with reported improved outcomes following CLM ablation, have made several investigators revisit the treatment paradigm to the role of surgery and ablation for the management of CLM patients.

In this review, we aim to discuss the current evidence on the management of patients with CLM and to provide up-to-date literature in regards to radiofrequency ablation (RFA) and microwave ablation (MWA), and irreversible electroporation (IRE) use and outcomes on such patient population. We will also expand our discussion on currently known gaps of knowledge on the liver ablation literature. Finally, taking this along with the available literature in respect to surgical resection for CLM, we will debate how such contemporary evidence can be utilized as guidance for appropriate decision-making.

## 1. Current evidence (the ‘known knowns’)

### 1.1. Patient selection: a key factor for optimal ablation outcomes

Liver ablation by either percutaneous or intra-operative approach has been traditionally utilized on patients with small CLM [2], with the aim of achieving local curative effect at the ablated lesion and prevent its local recurrence, also known as local tumor progression (LTP) [13]. For patients with extra-hepatic disease, liver ablation can be offered if all intra- and extra-hepatic diseases can be eradicated or if the extent of extra-hepatic metastasis is limited and not thought to be the main driver of prognosis [14–18]. Presently, the role of liver ablation in patients with extensive extra-hepatic disease is of unclear value. It has been proposed that patients with ≤ 5 liver tumors, preferably 1–3, should be considered as inclusion criteria for ablation [16,17,19–21]. Also, tumor size is associated with local tumor control and OS. Several studies have reported that the tumor size < 3 cm is associated with satisfactory local tumor control and with longer OS [15,17,21–26]. In the European Society of Medical Oncology guidelines [1], a maximum tumor diameter of ≤ 3 cm is recommended as it has been demonstrated to be an independent predictor of overall and LTP-free survival (LTPFS) [15,27–29]. To properly estimate tumor burden, patients who are being evaluated for liver ablation should have a recent cross-sectional study with intravenous contrast, such as computed tomography (CT), magnetic resonance imaging (MRI), or positron emission tomography-computed tomography (PET-CT), ideally within one month from the ablation procedure day.

**Figure 1.**
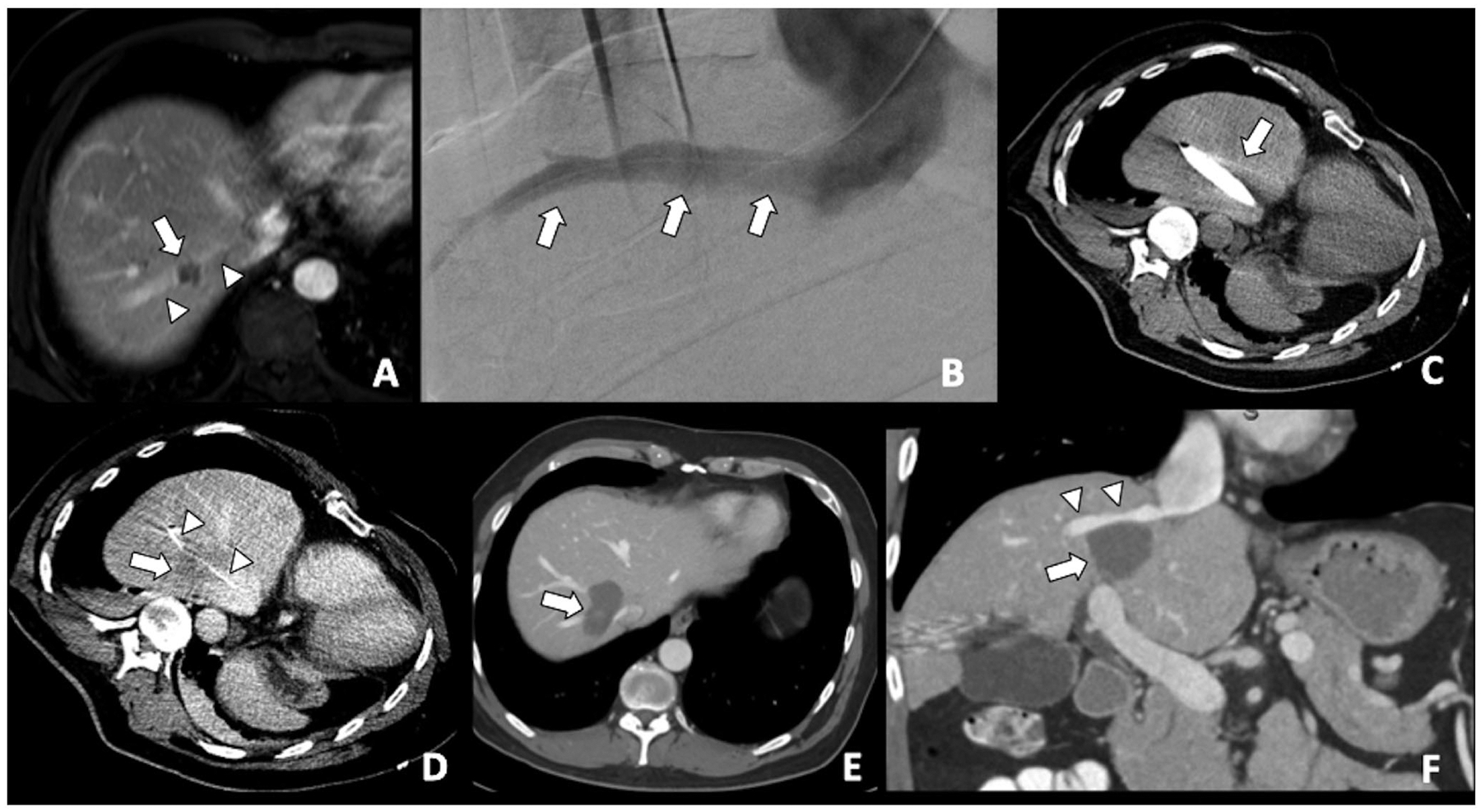
A colorectal liver metastasis abutting right hepatic vein treated by combination of percutaneous transhepatic venous temporary balloon occlusion of the right hepatic vein and microwave ablation to compensate the heat-sink phenomenon. (A) Magnetic resonance imaging demonstrating a tumor (arrow) abutting right hepatic vein (arrowheads); (B) Percutaneous transhepatic hepatic venography demonstrating the right hepatic vein (arrows); (C) Intra-procedural CT depicting inflated balloon catheter in the right hepatic vein during ablation; (D) A Clear ablation zone (arrow) covering the right hepatic vein, which is indicated by the catheter (arrowheads); (E) One month follow-up CT revealing satisfactory ablation zone (arrow) without residual tumor; (F) CT coronal multiplanar reconstruction demonstrating the ablation zone (arrow) and patent right hepatic vein (arrowheads).

Tumor location can affect both local tumor control and complication rates. Ablation of tumors adjacent to large vessels is associated with the heat-sink phenomenon and with an increased risk of residual tumor or LTP [30–32]. MWA is theoretically less susceptive to heat-sink effects than RFA, although this is still a relevant issue for large vessels with high flow rates [33–35]. The use of thermal ablation modalities to tumors adjacent to critical structures (i.e., major bile ducts, gallbladder, colon, pancreas, among others) can be associated with complications such as bleeding, vessel thrombosis, cholangitis, liver abscess, colon rupture, pancreatitis, among others [36–38]. Such complications can be avoided in a significant number of patients with percutaneous gas/hydrodissection techniques. In such situations, nonthermal ablation techniques such as irreversible electroporation (IRE) can be applied [39,40]. Because of the nonthermal nature of IRE, its efficacy is not impeded by the heat-sink effect by adjacent blood vessels [41]. Additionally, combined ablation and endovascular strategies such as combination with pre-ablation embolization or use of balloon-occlusion of the “adjacent vessel” responsible for heat-sink effect in combination with MWA (Figure 1) should be considered to compensate the heat-sink phenomenon. The influence of subcapsular tumor location on ablation effectiveness has conflicting results in the literature, with most of the authors showing similar LTP rates results when compared to nonsubcapsular-ablated tumors [42–45], whereas some other authors showing higher rates of LTP for subcapsular tumors [22].

More recently, the influence of tumor biomarkers on liver ablation outcomes has been reported [45–49]. RAS gene family (KRAS, NRAS, and HRAS) mutations are found in up to 40% of patients with colorectal cancer and portend a worse survival following resection of primary and CLM, more invasive tumor biology, and worse response to preoperative chemotherapy [50–52]. Also, RAS mutation is associated with a higher rate of positive margins after resection of CLM (11.4% mutant-RAS vs 5.4% wild-type RAS, *p* = 0.007) [53]. On a single-institution retrospective analysis of 92 patients who underwent ablation of 137 CLM, three-year LTPFS rates were significantly worse for mutant RAS patients compared to wild-type RAS patients (35% vs. 71%, *p* = 0.001) [45]. Similarly, on another single-institution retrospective analysis of 97 patients with CLM treated with RFA, KRAS mutation was a significant predictor for LTP of CLM ablated with margins 1 – 5 mm (LTP rates, 80% vs 41%, mutant vs wild-type RAS, respectively. *p* = 0.018) [47]. Table 1 provides a summary of current known patient’s factors associated with CLM ablation outcomes.

### 1.2. Technical factors: the importance of adequate imaging guidance and ablation margins

Effective ablation treatment relies on the success of four interdependent critical steps: tumor identification; ablation planning; probe placement according to the plan, and assessment of treatment delivery.

#### 1.2.1. Tumor identification

**Figure 2.**
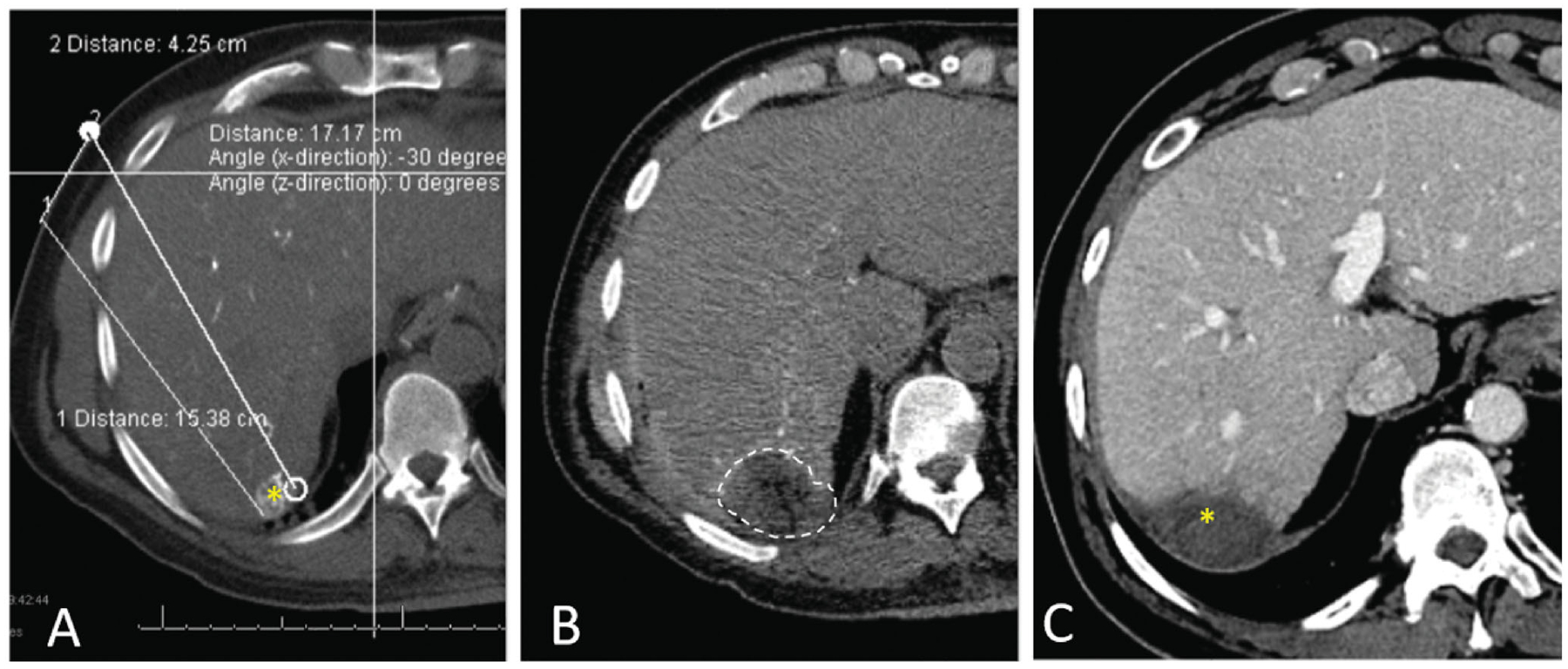
CT during hepatic arteriography (CTHA) on a patient with colorectal liver metastasis undergoing liver ablation. (A) CTHA prior to MWA antenna placement to localize target tumor (*); (B) postablation CTHA demonstrating large ablation zone (dotted line) around the treated tumor, which is no longer visualized; (C) 32-months CT follow-up demonstrating involution of the ablation zone without recurrence (*).

Several studies have demonstrated higher rates of LTP when only unenhanced imaging methods are utilized [14,54,55]. Therefore, the use of intravenous contrast-enhanced cross-sectional (CT or MR) imaging is preferred as it provides optimal tumor margins definition assessment [16]. More recently, the use of transcatheter CT during hepatic arteriography *via* the hepatic artery has been applied by some investigators for facilitating tumor identification and ablation endpoint assessment (Figure 2) [56,57]. This technique allows tumor identification by injecting smaller amounts of contrast media through the hepatic artery, and its use has been associated with improved local tumor control and superior LTPFS when compared to conventional CT fluoroscopy-guided ablation [56]. Another option to facilitate tumor identification is the use of image fusion, which allows the use of co-registered MRI/CT/PET datasets during ultrasound-guided or CT-guided interventions for ablation planning [58,59]. Also, a whole-body fluorodeoxyglucose (FDG) PET-CT scan can provide additional information for better quantification of liver and extrahepatic metastases and may change the management [60]. MRI is the most accurate imaging for the detection and characterization of hepatic metastases, especially with the hepatocyte-specific MRI contrast agent. It has a high sensitivity for the detection of smaller tumors that may not be easily detected by CT and PET [61].

#### 1.2.2. Ablation planning

To achieve sufficient ablation margins (>5 mm) around the tumor, careful planning of the procedure is the key. Recent studies indicated that ablation may provide acceptable oncologic outcomes for patients with small CLMs that can be ablated with sufficient margins [15,22,62]. One study has reported that margins 10 mm or larger are associated with no local tumor progression within a 24-month follow-up period [44]. The maximum size of the lesion that may be treated with one probe position depends on the short diameter of the expected ablation zone (which is related to the type of the ablation device and the liver perfusion at the tumor location). The *in vivo* short diameters of the ablation zone after using standard RFA and MWA probes range from 1.6 cm to a maximum of 3.6 cm [63,64]. Therefore, if the probe is perfectly placed in the lesion center the maximum tumor size that can be treated with one probe position ranges from 0.6 to 2.6 cm (depending on the type of probe/ablation system used). For larger lesions, multiple overlapping ablation zones have to be obtained, which can be facilitated by the stereotactic navigation system and 3 D-planning of probe trajectories which enable highly accurate probe positioning [65].

#### 1.2.3. Probe placement

**Figure 3.**
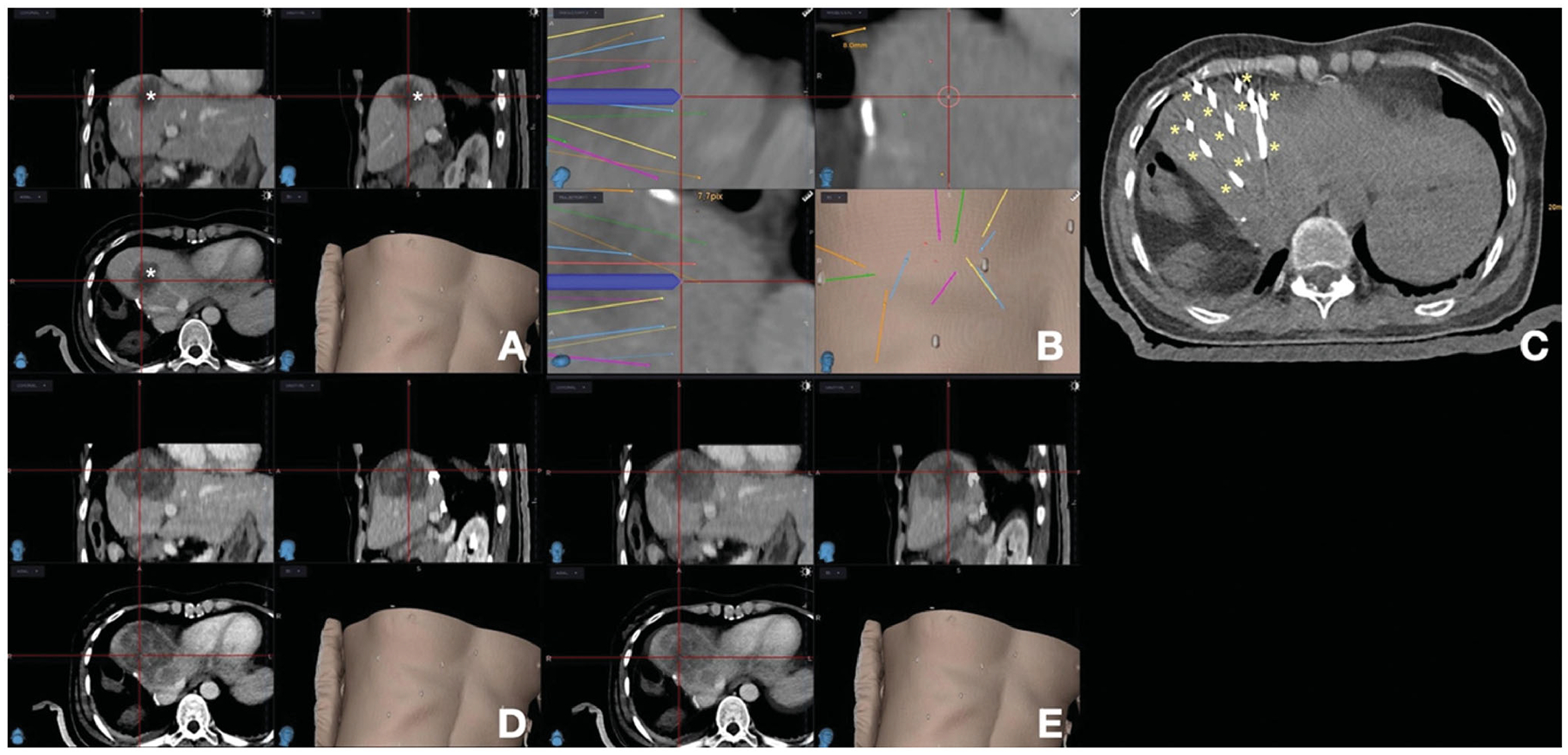
Stereotactic navigation systems on a patient with colorectal liver metastasis (CLM) undergoing liver ablation. (A) Posthepatectomy recurrent CLM measuring 5 cm in diameter (*); (B) Planning of 13 percutaneous guide needles trajectories, represented by different lines; (C) CT evaluation of guide needles placement (*); (D) Postablation CT for treatment verification. (E) Fusion of pre- and post-ablation CT for verification of ablation margin, showing complete tumor coverage with ablation with sufficient ablation margin.

Precise probe placement at the target tumor can be facilitated by stereotactic navigation systems that take into consideration tumor location, shape, size, as well distance between the ablation needles (Figure 3). Stereotactic radio-frequency ablation (SRFA) *via* 3 D imaging has been advocated by some authors as a reproducible method that allows improved local tumor control [65]. This is a commercially available, optical-based, frameless stereotactic navigation system combined with 3 D-planning software allowing for accurate planning of elaborate trajectories for single or multiple probes to cover an ablation zone with a clear margin [65]. On a retrospective analysis performed by Bale *et al*. [66], 63 consecutive patients submitted to 98 SRFA sessions for 189 CLM showed similar LTP rates between CLM <3 cm, 3–5 cm or >5 cm in diameter (17.7% vs 11.1% vs 17.4%, respectively. *p* = 0.6). 1-, 3- and 5-year survival rates were 87%, 44%, and 27%. Resectable patients had improved median OS rates when compared to unresectable ones (58 vs 27 months, respectively. *p* = 0.002). Based on such results, the authors concluded that SRFA can be considered as an alternative to surgical resection as a first-line treatment for CLM on an individual patient basis [66].

The percutaneous approach of ablation is usually favored over the surgical approach due to less post-treatment morbidity and shorter hospital stay. The reported major complications rates range from 3.1 to 4.4% for laparoscopic ablation, 9.6 to 32% for open ablation, and from 0 to 4.7% for percutaneous ablation [7,15,17,22,23,54,67–72]. A study of 233 patients with malignant hepatic tumors found shorter hospital stays and fewer complications of percutaneous RFA comparing to the surgical approach, although there was no significant difference in oncologic outcomes [73]. A meta-analysis has reported that there was no significant difference in 5-year overall and disease-free survival rates between percutaneous and open/laparoscopic approaches for ablation of CLM [74].

The role of anesthetic techniques in ablation is critical since it reduces the pain, anxiety, and patient’s movements during the procedure, therefore facilitating tumor targeting and potentially improving ablation outcomes [75]. Several anesthetic methods are used, such as general anesthesia, and sedation using fentanyl, midazolam, or propofol. General anesthesia is preferred because of controllable respiration during the procedure. Adjunctive methods such as intermittent breath-hold during probe placement, low tidal volume settings, and high-frequency jet ventilation can also be applied with that intent [16,27,76]. Precise respiratory triggering is mandatory if stereotactic techniques are used [66]. On a recent retrospective analysis by Puijk *et al* of 90 patients submitted to 114 ablation procedures under general anesthesia (*n* = 22), midazolam (*n* = 32), or propofol (*n* = 60), authors reported that sedation with propofol and general anesthesia was associated with better local tumor control than sedation with midazolam, providing local tumor progression rates of 4.3%, 5.7%, and 45.2%, respectively (*p* < 0.001) [77].

#### 1.2.4 Ablation assessment.

**Figure 4.**
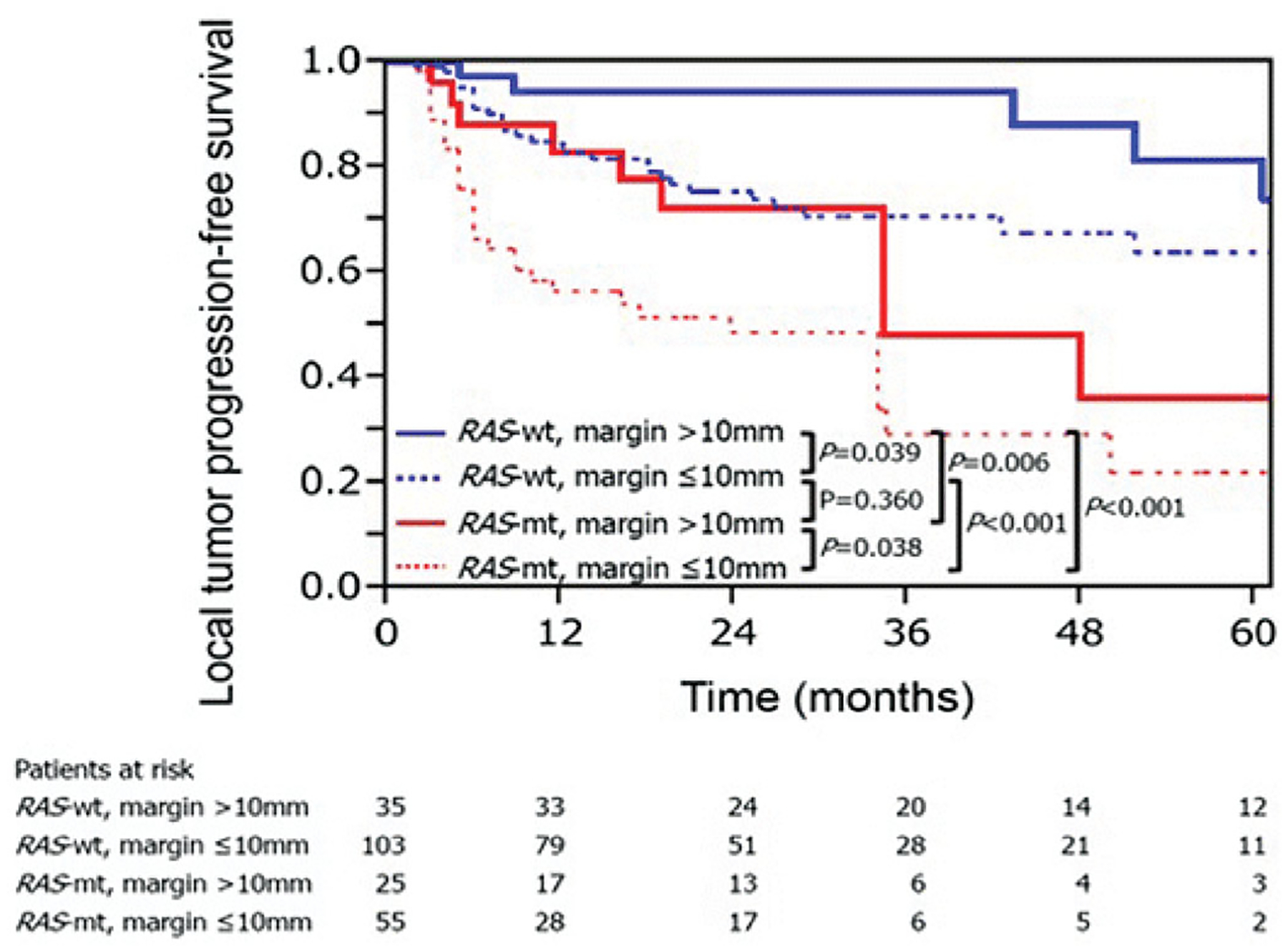
Kaplan–Meier curves for local tumor progression-free survival according to RAS and minimal ablation margin. RAS-wt: RAS wild-type; RAS-mt: RAS mutant. Adapted from Calandri M, Yamashita S, Gazzera C, Fonio P, Veltri A, Bustreo S, et al. Ablation of colorectal liver metastasis: Interaction of ablation margins and RAS mutation profiling on local tumor progression-free survival. Eur Radiol. 2018;28(7):2727–2734. Used with permission.

Sufficient minimal ablation margins are considered an essential technical factor for reducing rates of local tumor progression [15,22,24,44,62]. A minimal ablation margin of > 5 mm is considered essential for achieving acceptable local tumor control rates, which have been reported to be around 15%. It has been reported in two studies with long-term follow-up periods that ablation margin smaller than 5 mm was an independent risk factor for LTP [15,22]. Moreover, if minimal ablation margins are > 10 mm, LTP rates fall significantly to 0–5%, therefore making it the desired treatment endpoint for CLM ablation as it is on par with marginal recurrence rates after resection of CLM [11,15,16,44,62,78,79]. Similar to the surgical literature, the interplay between minimal ablation margins and tumor biology has also been recently evaluated for thermal ablation. Calandri *et al*. [46] have demonstrated on a two-institution retrospective study of 136 patients with 218 ablated CLM that three-year LTPFS were significantly worse in mutant than in wild-type RAS in both CLM subgroups with minimal ablation margin ≤ 10 mm (29% vs. 70%, *p* < 0.001) and >10 mm (48% vs. 94%, *p* = 0.006), respectively. Such results demonstrate that even when minimal ablation margins > 10 mm are achieved, LTP among mutant and wild-type RAS patients were still significantly different (Figure 4). Therefore, the authors concluded that ablation margins > 10 mm are crucial for mutant RAS CLM [46].

**Figure 5.**
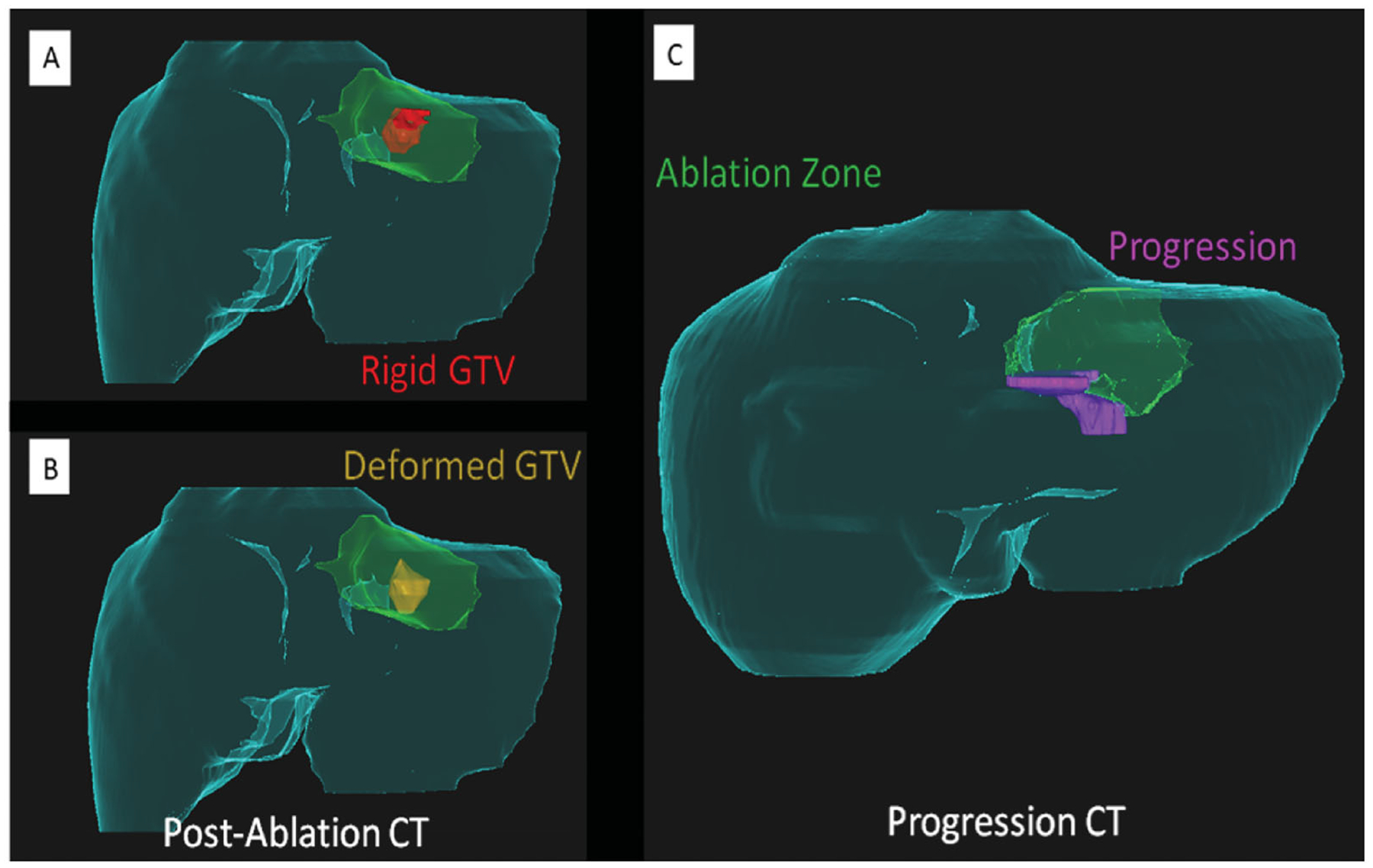
Importance of a deformable registration model use for assessing ablation margins. (A) 3 D CT reconstructionu using a rigid registration model. Gross tumor volume (GTV, red) is depicted onto the postablation CT maps within the ablation zone (green area); (B) 3 D CT reconstruction using a deformable registration model depicting GTV (yellow) in close contact with the ablation zone margin (green area) caudally, reflecting insuficient ablation margins (2 mm) at that region; (C) 6-month postablation 3 D CT reconstruction using a deformable registration depicting local tumor progression (purple area) and the caudal aspect of the ablation zone (green area), which was deemed to have insufficient ablation margins per biomechanical deformable registration model. Courtesy: Brian Anderson, PhD and Kristy Brock, PhD.

Despite the widely recognized need for sufficient minimal ablation margins on a three-dimensional plane, fundamental limitations still exist on how to properly assess such ablation margins intra- and post-procedurally. This is due to a series of factors: the tumor is obscured by the ablation zone, precluding its mapping within the ablation zone; the complex changes in the patient’s position, post-ablation tissue retraction and inflammation, and imaging resolution [80,81]. Ongoing efforts such as the use of deformable imaging registration methods aim to provide accurate modeling of ablation zone assessment (Figure 5) [80,82–86]. Furthermore, some intraoperative techniques are used to facilitate the targeting of tumors and ablation assessment. A split-dose technique for FDG PET/CT guidance has been developed, which allows prompt tumor targeting and immediate postablation assessment [87–89]. The main concept of this technique is that a smaller first dose of FDG before the ablation will be significantly decayed by the time the second larger dose is administered, allowing for the detection of FDG activity within any residual viable tumor. Another technique using intraprocedural nitrogen 13 ammonia perfusion PET has been developed to assess the ablation margins [86]. For ultrasound guidance, using intravenous contrast can improve tumor detection sensitivity [85,90]. A post-ablation contrast-enhanced ultrasound can provide an immediate evaluation of residual tumors and guidance to supplementary ablation [90].

Besides imaging assessment, intraprocedural pathology assessment can provide clinical information that has prognostic significance for LTP. The identification of viable Ki-67-positive tumor cells from the tissue fragment adherent to electrodes or tissue obtained from the biopsy of the tumor center and the suspected minimal margin of the ablation zone has been reported to be associated with LTP and OS [91–93]. A prospective study performed a biopsy of the ablation zone showing minimal margins <5 mm were likely to have biopsies with viable tumor cells (*p* = 0.019) [91]. In addition, immediate fluorescent assessment of ablation zone [94] or *in vivo* diffuse reflectance spectroscopy [95] has shown to be feasible for real-time identification of residual viable tumor cells.

### 1.3. Longitudinal sequential local therapies: a must from the get-go

In recent years, a shift in the oncological liver surgery approach of patients with CLM has occurred. The previous approach of performing major hepatectomies for wider tumor clearance and resultant ‘less at-risk liver’ has been superseded by the parenchymal-sparing hepatectomy (PSH) [96–99]. Such change has been driven by several reports showing that major hepatectomy is more frequently associated with postoperative liver insufficiency, while it does not prevent intrahepatic recurrence or improve survival [100]. Moreover, growth factors and cytokines, which might get elevated depending on the extent of liver resection, may also act as pro-oncogenic factors, ultimately promoting tumor progression [101–103]. Finally, it is known that up to 75% of the patients with CLM submitted to hepatectomy as the initial local curative modality will unfortunately present with recurrence during their disease course, with the liver representing a significant proportion of such recurrences [78,104,105].

In this scenario, allowing sufficient liver remnant following first local liver curative therapy might allow further application of local therapies such as re-resection or liver ablation at the time of recurrence, which has been shown to positively impact patient outcomes [71,98,106,107]. On a retrospective analysis of 300 solitary CLM patients, Mise *et al*. [98] showed that patients undergoing PSH, when compared to non-PSH patients, did not have worse OS, recurrence-free, and liver-only recurrence-free survival. More importantly, repeat hepatectomy was more frequently performed in the PSH group (68% vs 24%, *p* < 0.01) and, among patients with liver-only recurrence, better 5-year OS from initial hepatectomy and liver recurrence was shown on the PSH group versus the non-PSH. Finally, no difference in OS was noted after liver recurrence between patients who received repeated resection versus ablation at the time of recurrence (*p* = 0.143) [98].

Also, subsequent ablation to LTP after ablation could improve the survival compared to LTP who did not undergo re-treatment. Solbiati *et al*. reported that the median OS rates of patients undergoing repeated RFA for LTP and who did not re-treated were 45.5 months and 31.0 months, respectively (*p* < 0.001) [108]. Another study from Sofocleous *et al*. reported that patients undergoing repeated RFA for LTP had a significantly prolonged 3-year survival than those who didn’t (89% vs 23%, *p* = 0.03) [71]. These results support percutaneous thermal ablation as a longitudinal sequential local therapy for salvage treatment after disease recurrences.

Recent publications on the use of liver ablation for recurrent CLM after hepatectomy have shown LTP rates ranging from 5.1% to 16.7% [106,109,110]. On a retrospective analysis, Schullian *et al*. [109] demonstrated their experience with multi-probe stereotactic RFA in 64 patients with a total of 217 ablated CLMs. Despite the inclusion of patients with up to 25 metastases (median, *n* = 2) with diameters of up to 7.5 cm (median, 2.7 cm), LTP rate of 11.5%, median OS of 33.1 months, with 1-, 3- and 5-year OS rates of 90.1%, 46.2% and 34.8% after the first SRFA were achieved. Of the 25 tumors with local tumor progression, there were 13 (52%) tumors with size ≥ 3 cm and 3 (12%) with size ≥ 5 cm. Notably, 31 patients (48.4%) developed distant tumor recurrence away from the prior ablation of whom 15 patients received repeated SRFA [109]. Such results highlight the need for planning for sequential therapies on the longitudinal care of such patients and the ability to achieve OS rates with ablation similar to re-resection.

### 1.4. Ablation: standing against (and side-to-side) with resection

**Figure 6.**
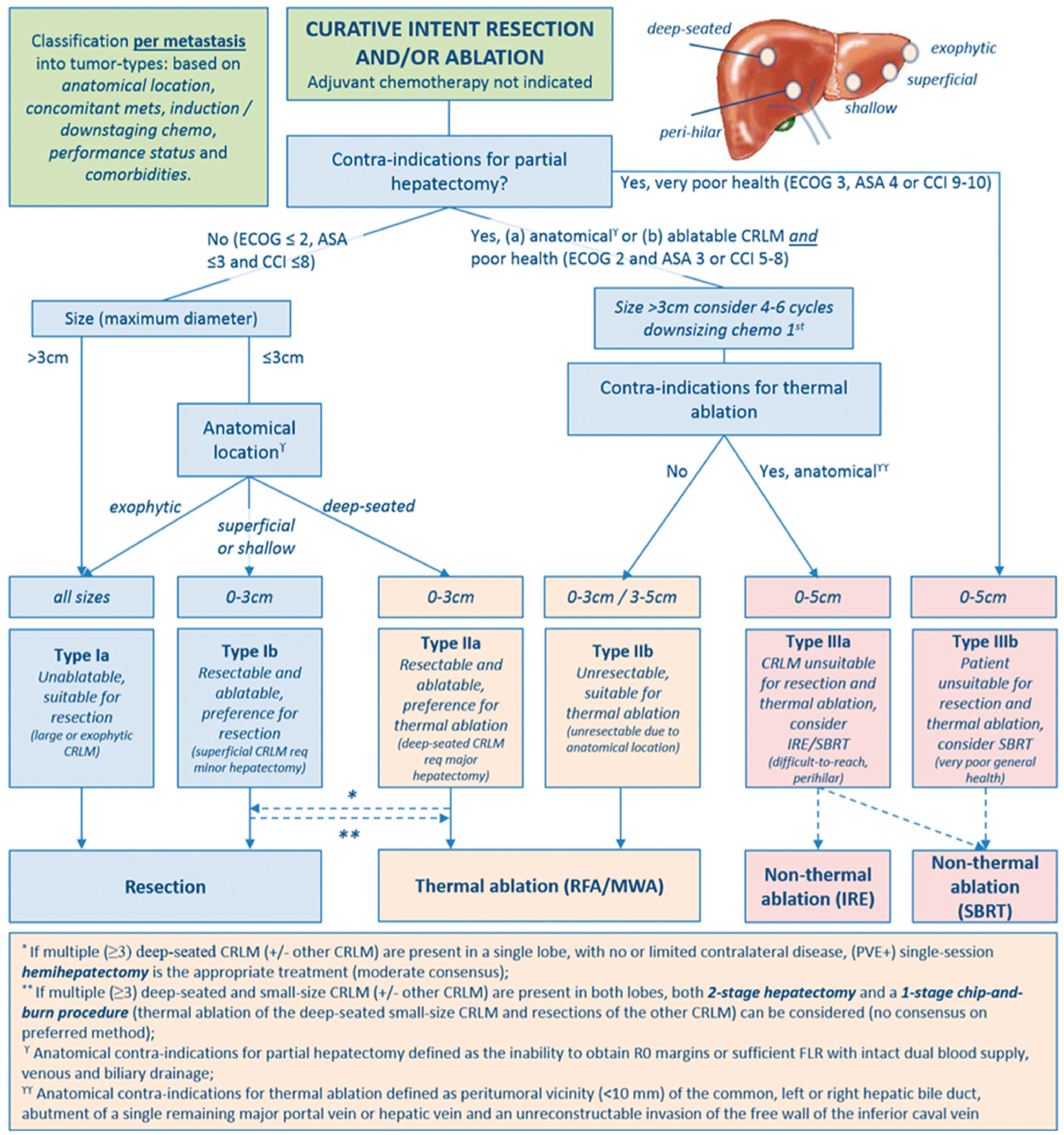
Assessment flowchart per-tumor of COLLISION trial. Adapted from Nieuwenhuizen S, Puijk RS, van den Bemd B, *et al*. Resectability and Ablatability Criteria for the Treatment of Liver Only Colorectal Metastases: Multidisciplinary Consensus Document from the COLLISION Trial Group. Cancers (Basel). 2020;12(7):1779. Published 2020 Jul 3.

**Figure 7.**
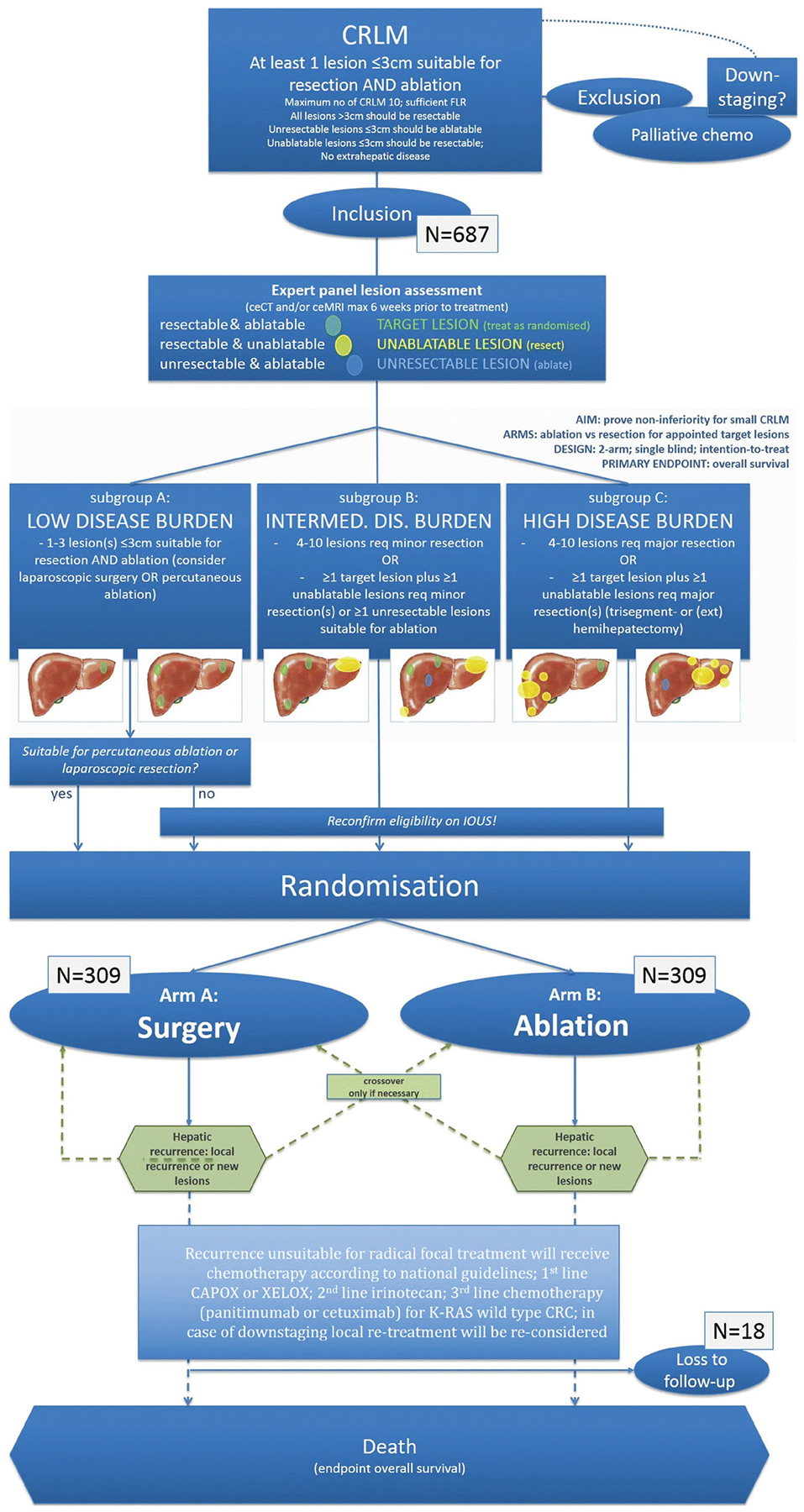
Flow diagram of study procedure from COLLISION trial. Adapted from Puijk RS, Ruarus AH, Vroomen LGPH, et al. Colorectal liver metastases: surgery versus thermal ablation (COLLISION) - a phase III single-blind prospective randomized controlled trial. BMC Cancer. 2018;18(1):821. Published 2018 Aug 15.

With its minimally invasive approach, faster recovery, superior safety profile, and reduced costs when compared to surgical resection, percutaneous ablation is an appealing treatment alternative for patients with small CLM. Ablation combined with systemic chemotherapy has been shown on a prospective phase II study to significantly prolong the OS in patients with unresectable CLM when compared to the use of chemotherapy alone [111]. Despite such potential advantages, to date, there are no available prospective randomized studies providing a definitive answer to this matter [4–8]. Retrospective non-randomized studies have reported that RFA has similar outcomes to resection with 5-year survival rates up to 55% [112–116]. A comparison of the CLOCC and EPOC trials showed that ablation had similar outcomes to resection for small tumors, which made authors support the use of RFA for local control in patients with limited metastases [29]. Contrarily, several meta-analyses have reported that liver resection was significantly superior to RFA in respect of overall and disease-free survival, although RFA showed a significantly lower rate of complications [8,74,117,118]. Recently, a case-matched study of 271 patients reported no difference in 3-year OS between patients submitted to MWA and hepatectomy as the first intervention for CLM (76 vs. 76%; *p* = 0.253) [119]. An ongoing noninferiority phase-III single-blind prospective randomized controlled trial comparing thermal ablation versus liver resection for CLM (the COLLISION trial) will add much needed evidence on the role of ablation as a first local therapy option [120]. In this trial, patients with at least one resectable and ablatable CLM (≤ 3 cm) and up to ten lesions are considered eligible (Figures 6 and 7). Additional unresectable tumors should be ≤ 3 cm and ablatable and additional unablatable tumors should be resectable. Patients undergoing any surgical resection or focal ablative liver therapy, systemic treatment ≤ 6 weeks before the procedure, or extra-hepatic disease are excluded. The primary endpoint is OS. The main secondary endpoints are disease-free survival, time-to-progression, primary and assisted technique efficacy, mortality, length of hospital stay, assessment of the quality of life, and cost-effectiveness [120].

The combination of hepatic resection with ablation has been proposed to achieve cure and preserve future liver remnants for patients with an extensive distribution of CLM [121–123]. Studies have reported that combined therapy of hepatic resection and ablation had comparable survival compared to hepatic resection alone [118,121,124]. Nevertheless, a study compared two-stage hepatectomy to one-stage hepatectomy combined with RFA for bilobar CLM and found an improved 5-year OS rate (35 vs. 24%; *p* = 0.01) and a lower incidence of postoperative hepatic insufficiency (6% vs 28%, *p* < 0.0001) in patients undergoing two-stage hepatectomy [125]. The authors inferred those surgeons may inadvertently over-ablate the lesions in an attempt to reduce the local recurrence rate but caused more unplanned damage to the liver remnant. Lastly, a new developed sequential treatment strategy, planned incomplete resection followed by postoperative percutaneous CT/MR-guided completion ablation for intentionally-untreated lesions, may provide better local tumor control when compared with intraoperative US-guided ablation (5-year local tumor recurrence: 31.7 vs. 62.4%; *p* = 0.03), while offering significantly lower rates of major postoperative complication [122]. Table 2 provides an overview of the most relevant ablation studies recently published.

## 2 Current gaps in knowledge (the ‘known unknowns’)

### 2.1. The relevance of co-mutations in liver resection

Although mutant RAS status of CLM has been already linked to worse outcomes after thermal ablation [45,46], it is currently unknown what is the interplay between co-mutations and ablation outcomes. RAS mutation in patients with wild-type TP53 and SMAD4 was not associated with a worse prognosis than wild-type RAS after CLM resection [126]. Additionally, the double mutations of TP53 with either KRAS, NRAS, or BRAF were associated with significantly worse survival compared with mutations in both gene groups alone [127]. Coexisting mutations in RAS, TP53, and SMAD4 were associated with significantly worse recurrence and survival than coexisting mutations in any 2 or 1 of these genes [126]. It is unknown that these genes with distinct signaling pathways can ‘cross-talk’ between each other or accumulate the hazard for survival.

### 2.2. The impact of prior hepatectomy

It has been reported that the history of prior liver resection is associated with improved local tumor control and survival of CLM thermal ablation. Odisio *et al*. reported that 3-year LTPFS (73 vs. 34%; *p* < 0.001), recurrence-free survival (23 vs. 9.1%; *p* = 0.026), and OS (78 vs. 48%; *p* = 0.003) were improved in patients with previous hepatic resection submitted to percutaneous ablation than on patients without previous hepatic resection submitted to percutaneous ablation [106]. Survival rates for patients submitted to liver ablation after hepatectomy in this series are in keeping with current survival rates reported after the first and second hepatectomy for CLM [128–130]. Although the patients with prior hepatic resection were younger, had ablation earlier on smaller CLM, and received less chemotherapy, the multivariable analysis did not disclose any of those factors associated with LTPFS. The authors suggested that the process of selecting patients for hepatic resection might have translated into factors positively affecting ablation outcomes of CLM who developed following resection. This might indicate the existence of currently unknown biological factors influencing ablation outcomes associated with patient selection for surgical resection. An ablation clinical risk score adapted from surgical clinical risk score [131], including the nodal status of the primary tumor, the time interval from primary resection to CLM diagnosis, number of tumors, and size of the largest tumor, is associated with local tumor control and OS [15,71,132]. This also has similar impacts on outcomes of ablation to the history of prior hepatectomy. Such observations further rebuke retrospective comparison of local recurrence rates between surgically resected and ablated patients, since such patient populations might be fundamentally different.

### 2.3. Influence of micrometastatic disease

The detection of circulating free tumor DNA, plasma microRNA, and circulating tumor cells, which are encompassed by the term ‘liquid biopsy’, is currently used clinically for therapeutic guidance, especially for the tumors with intratumor heterogeneity and clonal heterogeneity such as colorectal cancers. The technique could supplement existing clinical tools by improving screening, early detection, staging, recurrence identification, and prediction of clinical outcomes after treatment. Serial monitoring of the levels of circulating free tumor DNA during treatments can provide early detection of early disease progression in patients undergoing anti-EGFR therapies and immune-checkpoint inhibitors [133–136]. Similarly, the circulating tumor cells might represent a predictive biomarker of early response to chemotherapy and targeted agents [137,138], which might guide escalation or de-escalation of systemic treatment. Also, a challenge in CLM treatment is the development of resistance to systemic treatment, and ablation has been linked in some animal studies with aggressive tumor biological changes and tumor growth promotion [139,140]. It has been reported that liquid biopsy can detect oncogenic mutations in plasma before radiologic progression [141,142]. The liquid biopsy may provide serial monitoring of tumor heterogeneity and evolution over time and guiding clinical decisions [143]. Despite its intriguing potential, the clinical practice of liquid biopsy on patients undergoing ablation is limited due to the lack of standardization of the test and clear demonstration of its clinical benefits to date.

### 2.4. Salvage local therapy after the first resection of colorectal liver metastases: the impact of histopathological growth patterns

The histopathological growth patterns (HGP) of hepatic tumors appear at the interface between the tumor border and surrounding liver parenchyma. This includes three distinct growth patterns: a desmoplastic, a pushing, and a replacement type. They have been suggested to have the potential to predict the oncological outcomes of CLM and validated as prognostic biomarkers in patients undergoing resection of CLM with a replicable scoring method in international consensus guidelines [144–148]. Studies of CLM patients undergoing hepatic resection have reported more recurrence, microscopic residual tumor, and worse OS in non-desmoplastic HGP than in desmoplastic HGP [146,149]. Additionally, a higher rate of intrahepatic only recurrence and local treatments with curative intent for recurrence after hepatic resection was reported in desmoplastic HGP, inferring patients with better prognosis can still take salvageable local treatments [149]. Although the tumors with nondesmo-plastic HGP exhibit features associated with aggressive cancer biology and worse outcomes after hepatic resection [150], its impact on oncological outcomes of ablation is unknown, warranting further investigation.

## Conclusions

The fast-pacing evolving understanding of ablation therapies technology, imaging guidance, tumor biology, and artificial intelligence has the potential to clarify the current gaps in knowledge and to bring to light variables that were completely overlooked in the management of patients with CLM. When taken together, such observations can fundamentally change our perspective on the use of liver ablation. Moreover, by standardizing ablation planning, monitoring, and immediate response assessment, complete ablation (A0) with sufficient margins (ideally > 10 mm) can be achieved, which translates into lower rates of LTP that are on par with current surgical literature.

Based on the contemporary literature, aggressive local therapy with ablation (and, once possible, in combination with surgical resection) is associated with prolonged OS in patients with unresectable CLM. In addition, ablation for salvage after CLM resection recurrence can be considered in selected patients as the first-line modality of choice given similar OS and local recurrence rates to re-resection, and faster patient recovery. Finally, the role of ablation as first-line local therapy in comparison to surgical resection will have to be examined once the results of the COLLISION trial are available. Nevertheless, it is expected that both ablation and resection will play a synergistic role in the management of such patients, further highlighting the need for multi-disciplinary care for this patient population.

## Figures and Tables

**Table 1.** Patient’s factors affecting oncological outcomes.

Factors	Clinical Evidence / Effects
Tumor Size	Larger tumors are associated with worse local tumor control and overall survival. Size up to 3 cm is associated with satisfactory local tumor control and associated with longer overall survival [1–11]
Perivascular tumors	Some reports showed tumors adjacent to large vessels are associated with the heat-sink phenomenon and with increased risk of local tumor progression with RFA. Some reported MWA was not as affected by heat-sink effect, with satisfactory ablation margins [2,3,5,9,10,12–15]
Subcapsular tumor location	Controversial. Some literature reported the subcapsular tumor location is an independent risk factor of local tumor progression but some showed similar results compared to non-subcapsular tumor [2,12,14,16–18]
Tumor number	Tumor number is associated with overall survival. It has been proposed that patients with ≤ 5 liver tumor, preferably 1–3, should be considered as inclusion criteria for ablation [6,8,11,19–21]
RAS Mutations	Mutant-type RAS is an independent predictor of worse local tumor progression and overall survival. Minimal ablation margins >10 mm should be aimed in these patients [10,18,22,23]
Embryonic origin of primary colon cancer	Liver metastases from right-side primary tumors are associated with worse survival after ablation [14,24,25]
Previous liver resection	Prior hepatectomy for CLM is associated with improved local tumor control [12,26,27]
Clinical Risk Score	The score includes nodal status of the primary tumor, the time interval from primary resection to CLM diagnosis, number of tumors, and size of the largest tumor. It has impacts on local tumor control and overall survival [1,11,28]

**Table 2.** Selected recently published relevant ablation studies.

Author / year	Type of study	Intervention	Approach	Number of patients/lesions	Mean/median tumor size (cm)	Mean/median tumor number	Median follow-up period in months	Local tumor progression rate in %	Complication rate in %	3 y OS in %	5 y OS in %	8 or 10 y OS in %
Ruers et al. / 2017 [111]	Prospective (Phase II Trial)	RFA ± resection	Percutaneous or surgical	60 / 237^d^	4^f^	4	116.4	15	n.s.	56.9	43.1	35.9 (8 y)
		Systemic treatment		59 / 280^d^	4^f^	5	116.4	n.s.	n.s.	55.2	30.3	8.9 (8 y)
Meijerink et al. / 2021 [151]	Prospective (Phase II trial)	IRE	Percutaneous or surgical	50 / 76^b^	2.2	1.2	23.9	38	40^h^	n.s.	n.s.	n.s.
Han et al. / 2021 [22]	Retrospective	RFA	Percutaneous	365 / 512^c^	5^f^	n.s.	43.1	24.6	1^i^	58	41	n.s.
Shi et al. / 2021 [24]	Retrospective	MWA	Percutaneous	210 / 505^c^	2.7	2.4	48	23.8	2.4^i^	53.3	32.9	n.s.
Kurilova et al. / 2020 [62]	Retrospective	RFA and MWA	Percutaneous	286 / 415^c^	n.s.	_3_^g^	31	45.2	7^i^	53	37	n.s.
Tinguely et al. / 2020 [119]	Retrospective	MWA	Percutaneous or surgical	82 / n.s.^d^	3^f^	n.s.	25.2	n.s.	5^i^	69.1	n.s.	n.s.
Fan et al. / 2020 [152]	Retrospective	RFA	Percutaneous	144 / 258^e^	2.6	5.1	28.6	7	16.7^h^	n.s.	27.1	n.s.
Zimmermann et al. / 2020 [153]	Retrospective	RFA	Percutaneous	23 / 29^e^	3^f^	1.3	26	3.4	0^i^	57	24	n.s.
Schullian et al. / 2020 [109]	Retrospective	RFA^a^	Percutaneous	64 / 217^e^	2.7	2	21	11.5	5.8^i^	46.2	34.8	n.s.
Wang et al. / 2020 [21]	Retrospective	RFA	Percutaneous	85 / 138^c^	5^f^	_3_^g^	30	32.6	4.3^i^	45.6	22.9	n.s.
Cornelis et al. / 2018 [154]	Prospective	RFA, MWA, and IRE	Percutaneous	39 / 62	1.6	_3_^g^	22.5	37.1	n.s.	n.s.	n.s.	n.s.
Schicho et al. / 2019 [155]	Retrospective	IRE	Percutaneous	24 / n.s.^d^	2	2	26.5	n.s.	n.s.	25	8.3	n.s.
Mao et al. / 2019 [156]	Retrospective	RFA	Percutaneous	61 / 114^e^	2.7	2	28.9	16.7	n.s.	n.s.	33	n.s.
van Amerongen et al. / 2019 [157]	Retrospective	RFA + resection	Surgical	18/ n.s.	2.7	3	28	n.s.	39^h^	43	n.s.	n.s.
Calandri et al. / 2018 [46]	Retrospective	RFA, MWA, and Cryoablation	Percutaneous	136 / 218^d^	1.8	n.s.	25.1	17	n.s.	n.s.	n.s.	n.s.
Hof et al. / 2018 [115]	Retrospective	RFA ± resection	Percutaneous or surgical	35 / n.s.	1.9	n.s.	36.1	4.3	n.s.	n.s.	49.2	n.s.
Odisio et al. / 2018 [106]	Retrospective	RFA, MWA, and Cryoablation	Percutaneous	49 / 59^e^	1.4	5^g^	28	6.1	n.s.	78	n.s.	n.s.
Dupré et al. / 2017 [158]	Retrospective	RFA, MWA, and IRE	Percutaneous or surgical	33 / n.s.^e^	2	2	36.2	n.s.	12.1^h^	30.4	n.s.	n.s.
Imai et al. / 2017 [159]	Retrospective	RFA + resection	Surgical	37 / n.s.	1.3 (RFA) 2.9 (resection)	2 (RFA) 5 (resection)	35.6	3.5	22^i^	n.s.	55.9	n.s.
Sasaki et al. / 2016 [160]	Retrospective	RFA + resection	Surgical	86 / n.s.	2.2	5	30.9	n.s.	n.s.	52.6	37.2	n.s.
Shady et al. / 2016 [15]	Retrospective	RFA	Percutaneous	162 / 233^c^	1.8	n.s.	55	48	7^i^	48	31	n.s.
Valls et al. / 2015 [161]	Retrospective	RFA	Percutaneous	59 / 91^e^	2.3	1.5	25.3	19	8^i^	65	22	n.s.
Solbiati et al. / 2012 [108]	Retrospective	RFA	Percutaneous	99 / 202^d^	4^f^	8^g^	53	11.9	1.3^i^	69.3	47.8	18 (10 y)

Abbreviations: IRE Irreversible electroporation; MWA microwave ablation; n.s. not state; OS overall survival; RFA radiofrequency ablation.

a Stereotactic radiofrequency ablation.

b The lesions were unresectable and unsuitable for thermal ablation.

c The lesions were unresectable or recurrent colorectal liver metastases.

d The lesions were unresectable colorectal liver metastases or refused surgery.

e The lesions were recurrent colorectal liver metastases after liver resection.

f Maximal diameter.

g Maximal tumor number.

h Overall complication.

i Major complication.
